# Sustainable and healthy diet index (SHDI) unveils regional differences in Europe and Northern Africa: findings from the SysOrg study

**DOI:** 10.1007/s00394-025-03793-9

**Published:** 2025-09-11

**Authors:** Lea Ellen Matthiessen, Beatriz Philippi Rosane, Laura Rossi, Liliana Stefanovic, Dominika Średnicka-Tober, Rita Góralska-Walczak, Carola Strassner, Friederike Elsner, Youssef Aboussaleh, Zakia Hindi, Hamid El Bilali, Patrizia Pugliese, Sinne Smed, Jørgen Dejgård Jensen, Susanne Gjedsted Bügel

**Affiliations:** 1 Department of Nutrition, Exercise and Sports, University of Copenhagen, Frederiksberg, Denmark; 2 Department of Food and Resource Economics, University of Copenhagen, Frederiksberg, Denmark; 3 Council for Agricultural Research and Economics – CREA, Rome, Italy; 4 Department Organic Food Quality, Faculty of Organic Agricultural Sciences, University of Kassel, Witzenhausen, Germany; 5 Department of Functional and Organic Food, Institute of Human Nutrition Sciences, Warsaw University of Life Sciences, Warsaw, Poland; 6 Department of Food, Nutrition, Facilities, FH Münster University of Applied Sciences, Muenster, Germany; 7 Faculty of Sciences, Ibn Tofail University, Kenitra, Morocco; 8 International Centre for Advanced Mediterranean Agronomic Studies (CIHEAM-Bari), Valenzano, Italy

**Keywords:** SysOrg, Sustainable development, Healthy and sustainable diets, Diet index, EAT Lancet diet

## Abstract

**Purpose:**

We developed a diet quality index based on the Planetary Health Diet (PHD) to assess healthy and sustainable diets. The index was applied alongside socio-demographic characteristics in five regions across Europe and North Africa.

**Methods:**

The Sustainable Healthy Diet Index (SHDI) was designed using existing and validated healthy diet indexes. A total of 2,210 respondents from five case study territories (CST)—Cilento (Italy), Copenhagen (Denmark), North Hessia (Germany), Kenitra (Morocco) and Warsaw (Poland)—completed a food-group frequency questionnaire. Reported consumption frequencies for 24 food groups were converted to grams to assess compliance with PHD recommendations. A higher SHDI score indicates a more sustainable and healthier diet, although the environmental and health impacts are not directly assessed but are estimated based on intake at the food group level.

**Results:**

Copenhagen and Warsaw showed the highest SHDI scores, indicating better adherence to the PHD. In North Hessia, males had significantly better diet quality than females (*p* < 0.001). In Poland, female respondents with higher income and education had significantly better diet quality (*p* < 0.05). Compared to national studies, some food group intakes were unexpected, for example, high legume consumption in Copenhagen and high meat intake in Kenitra. Despite the Mediterranean diet being typical for Cilento, vegetable intake there was low.

**Conclusion:**

Some regions, such as Copenhagen, Kenitra, and Cilento, show beneficial eating patterns, particularly high legume intake. However, meat remains overconsumed in most CSTs. Our study highlights the need for further research to promote cultural acceptance of healthier and more sustainable dietary habits, such as increasing vegetable consumption and reducing meat intake.

**Supplementary Information:**

The online version contains supplementary material available at 10.1007/s00394-025-03793-9.

## Introduction

Many people today follow diets that negatively affect their health, especially in high- and upper-middle-income countries [1]. These diets often rely heavily on animal proteins (e.g., red meats), refined grains (e.g., white bread, polished rice), and highly processed foods rich in added sugars, salt, and saturated fats [2]. At the same time, intensified food production systems and disproportionate amounts of animal-based foods endanger the environment and are a significant source of global greenhouse gas emissions [3].

These dietary habits are both unhealthy and unsustainable, as they fail to meet the needs of the present without compromising future generations [4]. In response, the United Nations introduced the Sustainable Development Goals (SDGs) by [5], aiming to end poverty and inequality while protecting the planet. Achieving these goals requires a shift toward healthier and more sustainable eating patterns. As Mason and Lang emphasise, society must reject “high-carbon, biodiversity-destroying, water-guzzling food” and promote a more responsible culture [6].

To support this transition, the EAT-Lancet Commission proposed the Planetary Health Diet (PHD) as a science-based framework for sustainable and healthy eating. This study uses the PHD as a benchmark to evaluate dietary patterns in relation to the SDGs, particularly goal 3: Good health and wellbeing. The PHD aims to reduce the risk of non-communicable diseases and premature mortality through balanced food group recommendations. Because animal-based foods contribute significantly to environmental degradation, the PHD emphasises a plant-rich diet [2]. We therefore assume that diets aligned with the PHD are both healthier and more environmentally sustainable.

Dietary indexes are useful tools for assessing diet quality [7]. However, many of these tools require detailed dietary intake data, often collected through extensive food frequency questionnaires (FFQs), food diaries, or 24-hour recalls [8]. In contrast, indexes based on shorter FFQs may provide a more feasible and less resource-intensive approach to evaluating dietary quality.

We have taken the global PHD as a starting point, using its reference values as intake benchmarks in our developed diet quality index (namely the SHDI) and aim to gain a deeper understanding of local dietary patterns. Scholars have identified associations between diet quality and sociodemographic factors, such as age, education and gender. In general, better diet quality is associated with higher age and education levels [9]. However, these relationships are not always consistent. Some studies report lower diet quality among older adults and highlight significant inter-individual variation [10].

Research has also examined the intake of specific healthy food groups, such as vegetables and fruits, and how factors such as age, income, and gender influence consumption [11]. Women tend to have better diet quality than men [9, 11]. Studies on sustainable eating patterns have found that women, middle-aged individuals, and those with higher education levels are more likely to choose sustainable food options [12]. Nonetheless, regional differences in diet quality persist, even within the same country [10].

Based on a food-group frequency questionnaire, we adapted existing healthy diet indexes [13, 14], and developed the SHDI using the PHD’s reference values, which consider both environmental and health outcomes. These reference values serve as indicators of better health and environmental sustainability, although these outcomes were not directly assessed in this study but estimated based on food group intake patterns. The PHD was chosen as a benchmark because it provides a globally applicable reference point. This was particularly important given the diversity of local food-based dietary guidelines (FBDGs) [7, 15–17] and traditional diets, such as the Mediterranean diet, which are not uniformly applicable across all case study territories (CSTs). As previous research has shown [7], aligning food group classifications across nationally differing FBDGs presents methodological challenges, further supporting the use of a globally consistent benchmark like the PHD. It is important to note that the SHDI does not directly measure health or environmental outcomes. Instead, these aspects are estimated based on food group intake patterns, using the PHD recommendations as proxies for healthfulness and sustainability.

Our aim is to analyse diet quality and its sociodemographic determinants across regions, identifying key gaps and opportunities for improvement. This study contributes to the growing body of knowledge needed to support the transition toward healthier and more sustainable diets.

## Materials and methods

Local data collectors gathered information as part of the international research project “Organic agro-food systems as models for sustainable food systems in Europe and North Africa” (SysOrg). The project explores pathways toward more resilient and sustainable food systems. To support this aim, the consortium developed a Household Level Survey (hereafter referred to as the SysOrg survey) as the core data collection tool.

### The SysOrg survey

The survey consisted of four thematic sections: socio-demographic characteristics, dietary composition, organic food consumption, and food waste. This study focuses on socio-economic variables (i.e., age, gender, income, and education level) and dietary composition, assessed through a food frequency questionnaire (FFQ) covering 24 food groups. Respondents were asked how often they consumed specific food groups (e.g., How often do you eat vegetables?), choosing from ten frequency ranging from “never” to “every time I eat” (see Supplementary Material (SM) 1).

The survey was distributed in five geographical regions across Europe and North Africa (see Table 1). It was conducted online between January and March 2022, using non-probability river sampling [18], with local adaptations as described by Peronti and colleagues [19].

**Table 1:** SysOrg household survey by region (case study territory), country and survey distribution method

Country	Italy	Denmark	Germany	Morocco	Poland
Case Study Territory (abbreviation)	Cilento (CI)	Copenhagen (CO)	North Hessia (KA)	Kenitra (KE)	Warsaw (WA)
Geographical boundary	Cilento Bio-district is located in the Campania Region in Italy, and includes 95 municipalities	Located in the Capital of Denmark and includes the Municipality of Copenhagen	The northern part of the federal state Hessia which includes the districts of Kassel and Werra-Meißner	The province Kenitra consists of five districts and includes three urban and 20 rural communes	The city of Warsaw
Language(s)	Italian	English, Danish	English, German	English, Arabic, French	English, Polish
Distribution	River sampling and face-to-face distribution	River sampling	River sampling, in-class student recruiting, newspaper announcements, University’s news page	River sampling, face-to-face and paper-written format	River sampling, Municipality webpage
Reason for additional distribution	River sampling did not result in the expected number of participants	–	–	Include people without internet access and/or reading and writing skills	–

The questionnaire was based on validated tools [20–22] and adapted to reflect local dietary cultures. It covered multiple dimensions, including diet composition, organic food consumption, and food waste [19]. The international, multidisciplinary SysOrg consortium developed the survey through three validation steps:

1. Expert review: Two English-speaking experts in survey design and content (sustainable diet, organic food, and food waste) reviewed the questionnaire to identify and correct issues such as leading or confusing questions.
2. Translation and cultural adaptation: Native speakers translated the survey into Italian, Danish, German, Arabic, French and Polish. Two independent translators reviewed each version. Six native-language experts in each CST ensured the survey was understandable and culturally appropriate. For example, food examples in the FFQ were adapted to reflect local consumption habits and dietary guidelines [7].
3. Pilot testing: Two regular consumers in each CST completed the survey to test clarity and usability

The survey was primarily distributed online via social media (e.g., LinkedIn posts, local Facebook groups, and messenger groups), mailing lists, and university websites. In two CSTs, local adaptations were necessary. In Kenitra, a mixed-methods approach was used, combining online distribution with face-to-face interviews and paper-based surveys in public markets to reach individuals without internet access or literacy. In Cilento, due to low online response rates, data were collected face-to-face from a representative sample.

It is important to acknowledge the potential for self-selection bias, particularly in regions where the survey was distributed digitally. Individuals with higher nutritional awareness or a stronger interest in health-related topics may have been more likely to participate, potentially skewing the SHDI scores upward. This bias may be especially pronounced in the urban and digitally connected areas (e.g., Copenhagen and Warsaw), where such individuals are more easily reached through online channels.

### Study population

**Fig. 1:**
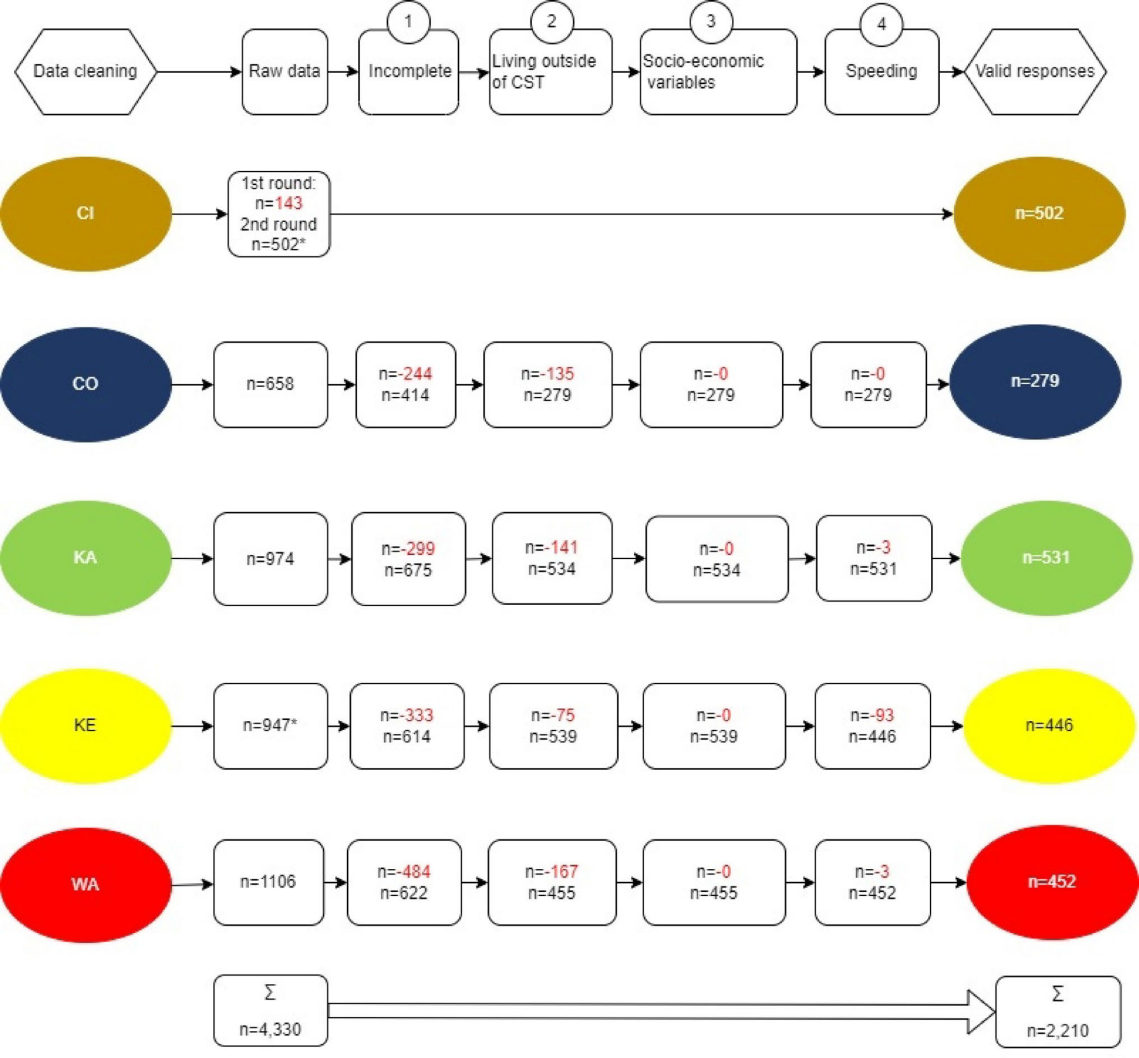
Decision Tree for the Data Cleaning of the SysOrg Household survey. *Note*: * indicates additional sampling methods (e.g. face-to-face and paper-written forms) were applied. For the data cleaning, red numbers indicate the number of respondents that were excluded in each step. Step 1) Participants who did not complete all five parts of the survey and step 2) who answered that they were not living in the geographical boundaries of the case study territories (CSTs) were excluded. Step 3) Participants under the age of 18 years or those who did not complete the socioeconomic questions regarding gender and income were excluded, but not if participants in terms of gender and income ticked the box “I do not prefer to answer”. Step 4) Participants were excluded based on the time of filling out the survey to exclude ghosts. The median from the total time (22 min) was taken and those participants who filled out the survey with less than half the median (< 11 min) were excluded

Figure 1 shows the decision tree used to determine the final sample. Of the 4,330 participants who began the survey, 2,210 valid responses were included. Respondents ranged in age from 18 to 95 years old. Exclusion criteria included incomplete responses, residence outside the CSTs, and unrealistically short completion times. The median completion time was 22 min; responses completed in less than 11 min (half the median) were excluded to ensure data quality.

### Development of the SHDI

The Sustainable Healthy Diet Index (SHDI) was developed to assess the healthiness and environmental sustainability of diets, using the Planetary Health Diet (PHD) [2] as a benchmark. The SHDI builds on existing indexes [13, 14] and was adapted to the FFQ used in the SysOrg survey. While the SHDI captures key elements of diet quality and environmental impact, it does not encompass all dimensions of sustainability. For example, the economic pillar (e.g., food affordability or security) is not included, and, social pillar is represented only through health-related aspects, such as dietary composition [23].

The PHD defines a “safe operating space” for food systems [2], a range in which diets can support human health without exceeding planetary boundaries. It provides scientific targets for food group consumption to reduce environmental impact and promote health [2].

Food groups in the SysOrg survey were categorised as either “adequate” or “limit” (see Table 2). “Adequate” groups are those with recommended intake levels in the PHD and are part of a healthy sustainable diet. “Limit” groups are those which should be minimised or not exceeded (e.g., sugars). Some food groups (i.e., dried fruits, refined grains, butter and margarine, cooking oil types) were excluded from the SHDI because they were not part of the PHD recommendations or contributed minimally to overall dietary impact.

**Table 2:** Sustainable Healthy Diet Index components and their equivalence in the SysOrg Households Survey, food groups' daily intake recommendations and reference values used for scoring diet quality

Intake category	Planetary Health Diet^a^	Reference values for the SHDI
*Adequate*		
Fruits	200 g (100–300)	200 g (100–300)
Vegetables	300 g (200–600)	300 g (200–600)
Legumes^b^	Cooked^c^ beans, lentils, and peas: 125 g (0–250) Soy foods 25 g (0–50)	150 g (0–300)
Nuts^bd^	Peanuts 25 g (0–75) Tree nuts 25 g	50 g (0–75)
Whole-grain cereal products	Cooked grains 580 g (0–60% of energy)	485 g (390–580)^f^
Potatoes	50 g (0–100)—potatoes and cassava	50 g (0–100)
Fish or shellfish^e^	28 g (0–100)	28 g (0–100)
Dairy products (milk, yoghurt, kefir, skyr, etc.)	250 g (0–500)	250 g (0–500)
Cheese	Included in dairy	20 g (0–20)^g^
Eggs	13 g (0–25)	13 g (0–25)
White meats^e^	29 g (0–58)	29 g (0–58)
Red meats^e^	14 g (0–28) Beef and lamb 7 g (0–14) Pork 7 g (0–14)	14 g (0–28)
*Limited*		
Sugary drinks	All sugars 31 g (0–31)	
Desserts and sweets	All sugars 31 g (0–31)	
Processed meats	Not recommended	
Alcohol	Not recommended	
Fast foods	Not recommended	
Processed salty snacks	Not recommended	

Following the EAT-Lancet recommendation, the reference values are expressed with a target value with a recommended intake range in brackets in parentheses (“minimum”- “maximum” intake)

^a^Recommendations for 2500 kcal/day[6]

^b^According to the EAT-Lancet [2], legumes, peanuts, tree nuts, seeds and soy are interchangeable. For this study, it was decided that legumes and soy should be separated from peanuts and tree nuts because of the difference in consumption patterns

^c^The PHD recommendations are for dry pulses and grains (dry beans, lentils, and peas 50 (0–100), dry grains 232 g) [2]; cooked weight estimated using a weight change factor of 2.5[24]

^d^Portion sizes were given by single nuts; for this study, we consider realistic portions, i.e. 3 Brazil nuts, 4 pecan nuts, 10 macadamias/cashew nuts/hazelnuts/pistachios/almonds[2]

^e^Both the recommendations and standard portions consider raw meats without bones, i.e. edible cut

^f^As energy intake was not available, a range of intake was required; based on values proposed in the Danish Adapted Healthy Plant-Based Diet[25, 26]

^g^The PHD does not specify a recommended intake for cheese; the values proposed in the Danish Adapted Healthy Plant-Based Diet [25] were used

A higher SHDI score indicates a more sustainable and healthier diet. However, the environmental and health impacts are not directly measured but are estimated based on intake at the food group level.

**Fig. 2:**
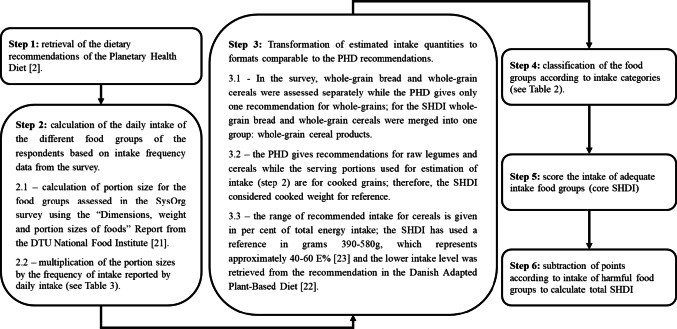
Flowchart overview of the development of the sustainable healthy diet index (SHDI)

The scoring for the SHDI was inspired by previously published diet quality indices (see SM 2), but it does not follow any single index exclusively, as existing models were not fully compatible with the data collected in this study. An overview of the SHDI development process is shown in Fig. 2.

To calculate the total SHDI score, two scoring strategies were applied. For food groups categorised as having an “adequate intake” (see Table 2), it was assumed that any intake has a health impact. Intake within the recommended range is considered beneficial, while intake below or above this range may be detrimental. Four scenarios were defined, each with a corresponding scoring equation:

(1) Below the recommended minimum: Intake below the recommended minimum ($${rec}_{min})intake$$ (but above 0) were scored using Eq. 1. It was used for intakes of fruits between 0 and 100 g ($${rec}_{min})$$, for example. In this scenario, intake between 0 and the recommended minimum is scored between 0 and 5.

1 $$ \max \left( {0 ;5 \times \frac{{intake - rec_{L} }}{{rec_{min} - rec_{L} }}} \right) $$

where intake = estimated reported intake, rec = target recommendation, ${rec}_{L}$ = intake level below which score is 0, determined as $\text{max}\left(0 ; {rec}_{min}-\left(rec-{rec}_{min}\right)\right)$

(2) Between the minimum and target recommendation: Intake between the minimum recommended intake ($${rec}_{min})$$ and the target recommendation (rec), Eq. 2. In the example of fruits, it was used in intakes between 100 g ($${rec}_{min})$$ and 200 g (rec).

2 $$ 5 + \left( {5 \times \frac{{intake - rec_{min} }}{{rec - rec_{min} }}} \right) $$

(3) Between the target and maximum recommendation: Intake between the target recommendation and the maximum recommended intake ${rec}_{max}$ is scored by Eq. 3. In the case of fruits, for intakes between 200 g (rec) and 300 g (${rec}_{max}$).

3 $$ 5 + \left( {5 \times \frac{{rec_{max} - intake}}{{rec_{max} - rec}}} \right) $$

(4) Above the maximum recommendation: Intake that surpasses the maximum recommended intake is scored by Eq. 4. For fruits, that is for intakes between 300 g ${(rec}_{\text{max}}$) and 450 g (${rec}_{U}$).

4 $$ \max \left( {0 ; 5 \times \frac{{rec_{U} - intake}}{{rec_{U} - rec_{max} }}} \right) $$

where upper excessive intake ${rec}_{U}$ stands for a cut-off point for a score of 0, which is assumed to be equal to $1.5\bullet {rec}_{max}$.

The sum of scores for all “adequate intake” food groups formed the Core SHDI score.

In addition, the SHDI accounted for six food groups classified under “limited intake” (Table 2), where frequent consumption is considered harmful to health, [13, 14]. For these groups, points were subtracted from the total SHDI based on reported intake frequency, following the scheme in Table 3. The total SHDI score could range from 0 to 120, with up to 54 points deducted for high intake of the “limit” food groups.

**Table 3:** Options of frequency intake report in the SysOrg Survey and points subtracted for each option reported

Frequency of intake	Points subtracted
Never	0
Less than once a month	1
1–3 times per month	2
Once a week	3
2–4 times per week	4
5–6 times per week	5
Once a day	6
2–3 times per day	7
4–5 times per day	8
Every time I eat	9

### Statistical analyses

Core and total SHDI scores were calculated using Microsoft® Excel® for Microsoft 365 MSO (Version 2304 Build 16.0.16327.20200, 64-bit). To account for household size, reported household income was adjusted using the OECD equivalence scale, dividing income by the square root of the number of household members. This adjustment enabled meaningful comparisons across households of different sizes. Linear regression analyses were conducted to examine association between socioeconomic variables and total SHDI scores within each CST. The regression model was specified as follows:

5 $$ \begin{aligned} {\text{SHDI}}_{i} & = \upbeta _{0} + \upbeta _{{{\text{gend}}}} \cdot {\text{gender}}_{{\text{i}}} + \upbeta _{{{\text{age}}}} \cdot {\text{age}}_{{\text{i}}} + \upbeta _{{{\text{age}}}}^{2} \cdot {\text{age}}_{{\text{i}}}^{2} \\ & \quad + \upbeta _{{{\text{educ}}}} \cdot {\text{education}}_{{\text{i}}} + \upbeta _{{{\text{inc}}}} \cdot {\text{income}}_{{\text{i}}} \\ \end{aligned} $$

where ${\text{SHDI}}_{\text{i}}$ represents the individual’s total SHDI score, and the predictors include gender, age, education, and adjusted household income).

To assess differences in average food group intake across CSTs, a two-way ANOVA was performed. All statistical analyses were conducted using RStudio (version 4.2.1,2022-06-23 ucrt).

## Results

For the present study, we included 2,210 respondents, not evenly distributed among the five CSTs. Respondents from Copenhagen presented the diet closest to the PHD (Core SHDI score), while the highest total SHDI scores were observed in Copenhagen and Warsaw (Table 4). The higher the SHDI score, the more sustainable and healthier the diet.

**Table 4:** Characteristics of respondents by mean total SHDI score and socioeconomic variables (age, gender and education) of the SysOrg Survey per case study territory

	All CSTs	Cilento	Copenhagen	North Hessia	Kenitra	Warsaw
Total respondents (n)	2210	502	279	531	446	452
Core SHDI score (mean ± SD)	57 (± 9)	56 (± 8)^a^	61 (± 8)^b^	59 (± 7)^b^	50 (± 10)^c^	59 (± 8)^b^
Total SHDI score (mean ± SD)	42 (± 12)	41 (± 11)^d^	46 (± 9)^e^	44 (± 9)^e^	35 (± 14)^f^	45 (± 11)^e^
Points deducted from harmful food groups (mean ± SD)	15 (± 6)	15 (± 6)^g^	15 (± 5)^g^	14 (± 5)^g^	15 (± 7)^g^	14 (± 5)^g^
Age (years) (mean ± SD)	44 (± 16)	53 (± 17)^h^	44 (± 15)^i^	47 (± 16)^j^	35 (± 11)^k^	39 (± 13)^l^
Gender (females, (n %))	1250 (57)	260 (52)^m^	233 (83)^n^	162 (30)^o^	222 (50)^m^	373 (82)^n^
High education level (n %)^1^	1252 (57)	118 (23)^p^	242 (87)^q^	289 (54)^u^	244 (55)^v^	359 (79)^q^
High disposable net household income, (n %)^2^	414 (19)	11 (2)^w^	115 (41)^x^	115 (22)^y^	13 (3)^z^	160 (35)^aa^

^1^Bachelor’s degree, master’s degree, PhD, or equivalent

^2^Number of respondents who reported having the two highest income levels available for disposable net household income, not adjusted for household size; values for each country are available in SM 3. The letters represent the results from post-hoc analysis with Tukey method, where the same letter in several CSTs indicates similar distribution (*p* value > 0.05) of the social characteristic

### Studied population

The studied samples consisted mainly of middle-aged females with higher education and high-income levels (Table 4). However, the characteristics of the SysOrg Survey respondents varied between the CSTs, as did their average SHDI total scores (Table 4).

**Fig. 3:**
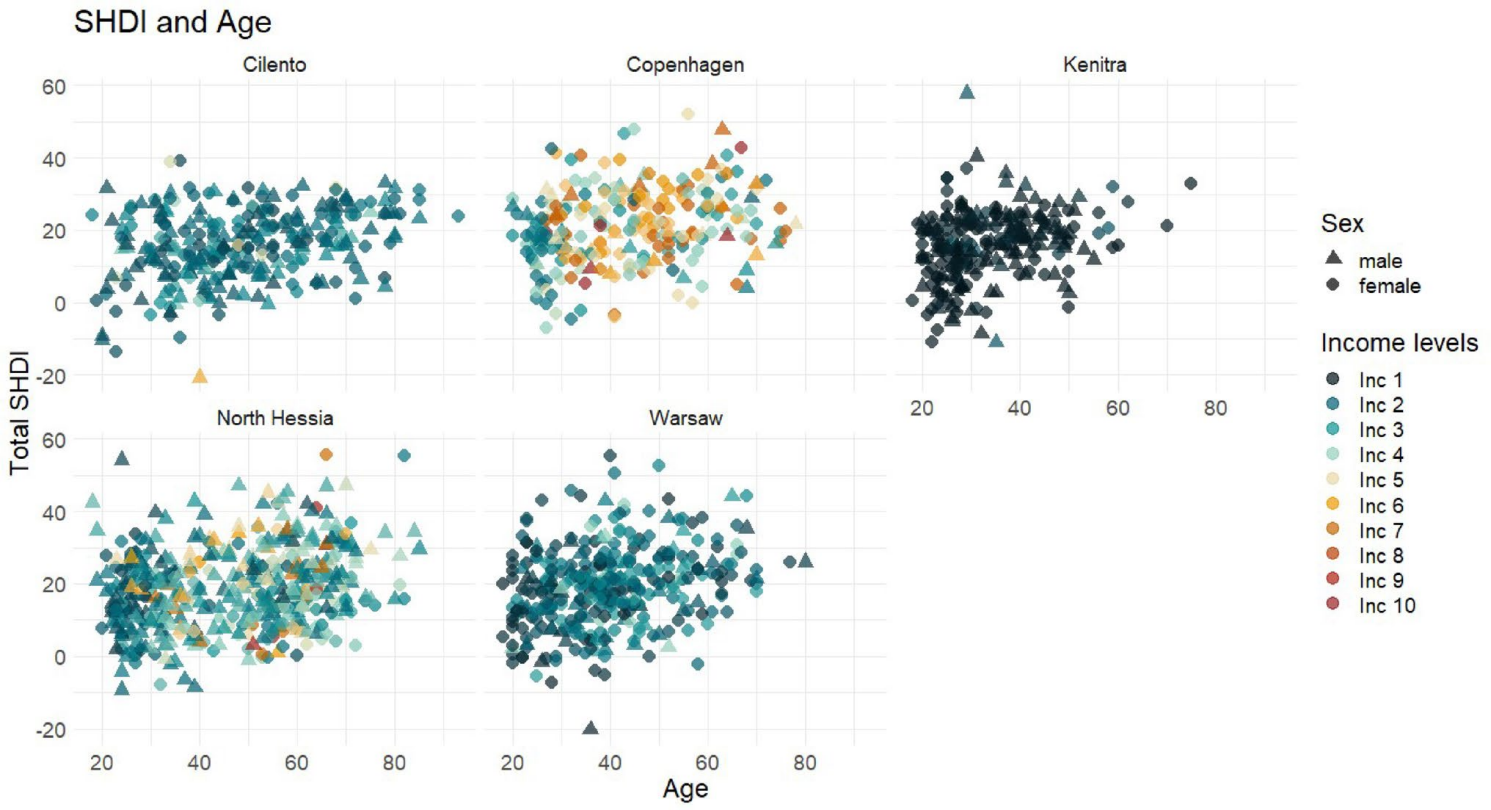
Scatterplot for total SHDI (y-axis) and respondents' age in years (x-axis). *Note*: Income levels represent all household adjusted income grouped into increasing ten levels, where “Inc 1” is the lowest

The distribution of household-size adjusted income was most diverse in CSTs Copenhagen and North Hessia (Fig. 3). In Kenitra, there is the lowest variation in the population in both age, income and diet quality, and the opposite is true for Cilento (Fig. 3).

### Diet quality and sociodemographic characteristics

In the investigation of associations between diet quality (i.e. total SHDI) and sociodemographic characteristics, it was observed that diet quality increased with age (Table 5, model 1). Diet quality differs between genders: In North Hessia, males had a higher total SHDI compared to females, while females had healthier diets in Cilento and Warsaw. Higher income was associated with a higher total SHDI in Warsaw, while lower income was associated with a higher SHDI in Cilento. Higher education level was associated with better diet quality in Warsaw (Table 5).

**Table 5:** Summary of Significance levels by Case Study Territory and Socio-economic Variables (i.e. age, income, gender and education), showing coefficients and p-values associated with t-statistics in the multilinear regression models tested

	Intercept total SHDI		Gender		Age		Age^2^		Education		Income	
	Coef./std. error	p-value	Coef./std. error	p-value	Coef./std. error	p-value	Coef./std. error	p-value	Coef./std. error	p-value	Coef./std. error	p-value
Cilento												
Model 1	29.546/3.157	< 2e–16	2.990/1.256	0.018	0.244/0.040	4.29e–09			− 0.064/0.472	0.891	− 0.178/0.077	0.021
Model 2	32.672/5.627	1.8e–08	3.026/1.259	0.017	0.093/0.228	0.682	0.001/0.002	0.503	0.011/0.486	0.981	− 0.179/0.077	0.020
Copenhagen												
Model 1	41.401/3.606	< 2e–16	1.271/1.584	0.423	− 0.0003/0.041	0.995			0.914/0.534	0.088	− 0.335/0.026	0.199
Model 2	29.863/5.898	8.05e–07	0.594/1.592	0.709	0.680/0.279	0.016	− 0.007/0.003	0.015	0.646/0.539	0.232	− 0.047/0.026	0.076
North Hessia												
Model 1	43.013/1.907	< 2e–16	− 3.514/0.929	0.0002	0.017/0.027	0.530			0.379/0.307	0.218	− 0.008/0.024	0.736
Model 2	51.023/3.940	< 2e–16	− 3.704/0.928	7.64e–05	− 0.403/0.183	0.028	0.004/0.002	0.021	0.495/0.310	0.111	0.003/0.025	0.889
Kenitra												
Model 1	23.316/3.926	1.06e–08	0.370/1.423	0.795	0.280/0.078	0.0004			0.259/0.416	0.533	0.462/0.369	0.211
Model 2	15.647/7.811	0.046	0.810/1.474	0.583	0.713/0.389	0.068	− 0.005/0.005	0.257	0.221/0.417	0.596	0.456/0.369	0.59
Warsaw												
Model 1	27.026/2.938	< 2e–16	5.994/1.421	3.17e–05	0.108/0.0146	0.020			1.144/0.517	0.028	0.134/0.068	0.049
Model 2	33.779/5.023	7.71e–11	6.135/1.419	2.05e–05	− 0.319/0.262	0.224	0.005/0.003	0.099	1.407/0.540	0.009	0.151/0.068	0.028

Coef.: coefficient. Std. error: standard error. Model 1: Linear regression using Eq. (2). Model 2: Linear regression using Eq. (2) is displayed in the methods section with age^2^ to account for a potential non-linear effect of age on the SHDI. Income analyses were done with household size adjusted income in Euros divided by 1000, gender was tabulated using 0 for males and 1 for females, age was measured in complete years, and education is measured in degree levels from 0 (no formal education) to 7 (PhD degree or higher)

### Dietary recommendations versus observed intake

**Fig. 4:**
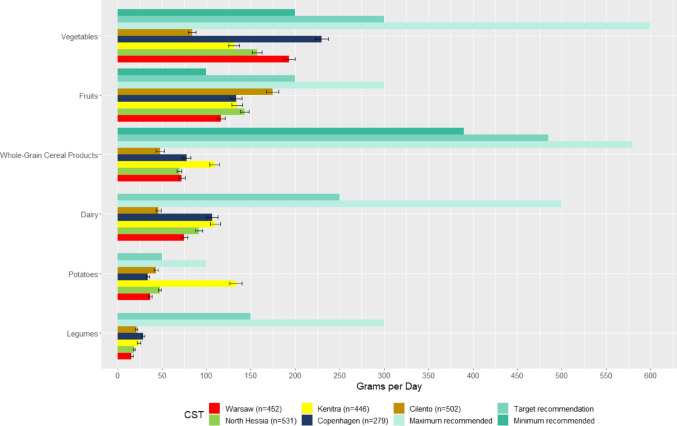
Recommended daily intake according to the Planetary Health Diet and mean estimated daily intake from participants of the SysOrg Survey for vegetables, fruits, whole-grain cereal products, dairy, potatoes and legumes

**Fig. 5:**
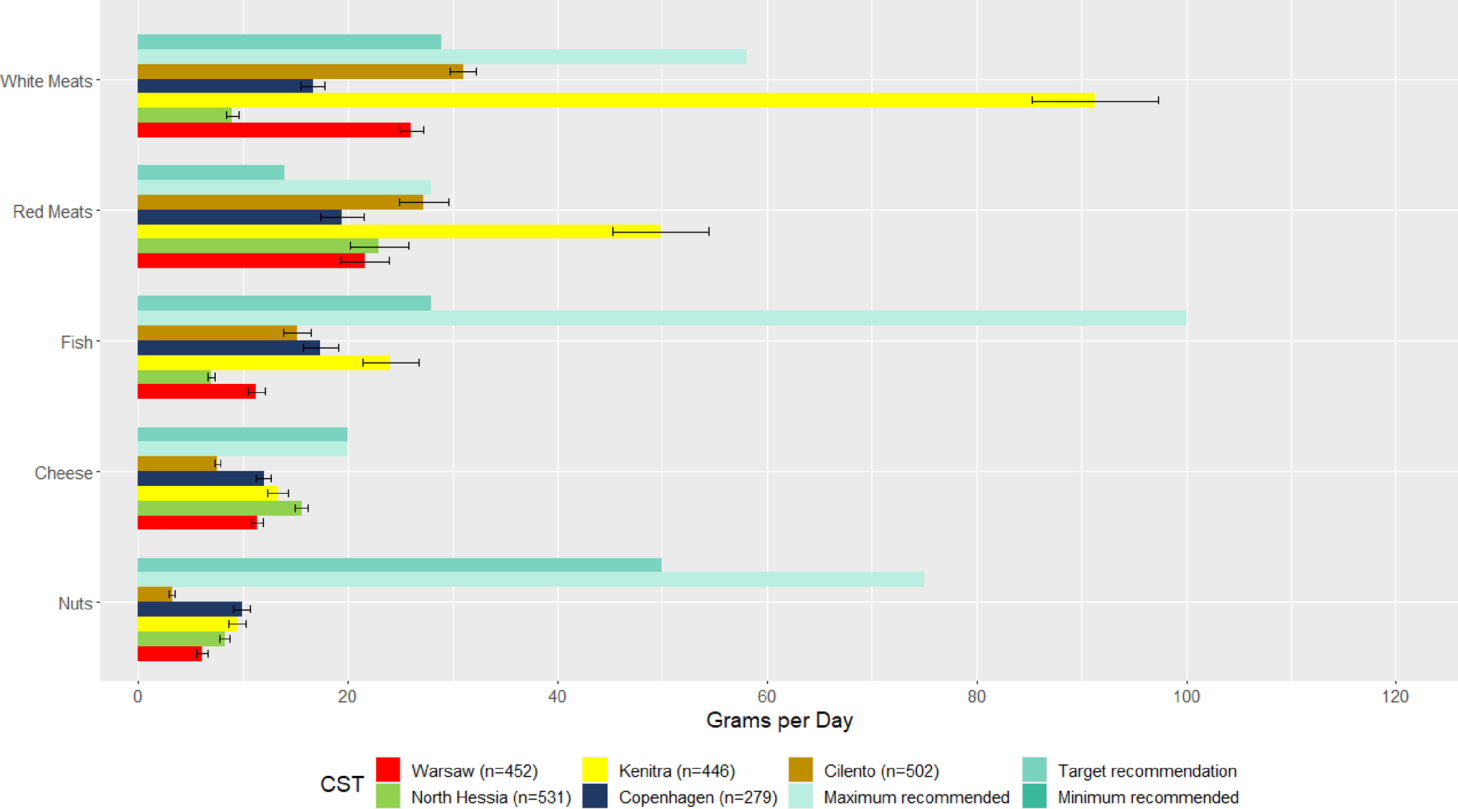
Recommended daily intake according to the Planetary Health Diet and mean estimated daily intake from participants of the SysOrg Survey for white meats, red meats, fish and shellfish, cheese and nuts

The reported intakes of the SysOrg population are not aligned with the PHD’s recommendations. All CSTs reported a lower intake of vegetables than the minimum recommended values, except for respondents in Copenhagen (Fig. 4). The food group of fruits is the only one where intake falls between the minimal and target recommendations. Average intake lower than the minimum recommendations was reported for whole-grain cereal products, dairy, legumes, fish, cheese and nuts (Figs. 4 and Fig. 5). Excessive intake of potatoes, as well as white and red meats, was observed in Kenitra.

### Dietary intake

**Fig. 6:**
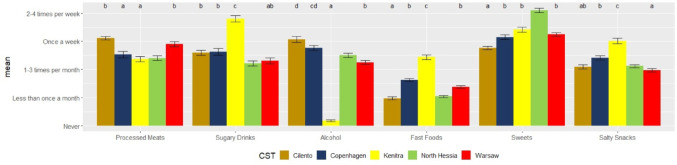
Frequency intake (y-axis) per food group (x-axis) of limited intake in the five case study territories (CSTs). *Note*: The letters on the top of the graph are the results of analyses of variance (similar letters indicate lack of statistically significant differences (*p* > 0.05) between CSTs within a food group) and the bars are standard deviations

In Cilento, the predominant consumption was noted for fruits and legumes, but also the least frequent intake of vegetables, nuts, whole-grain bread, and cereal products, compared to the other four CSTs (Fig. 4). Respondents in Cilento also reported the highest consumption of alcohol and processed meats but exhibited the lowest frequency of other unhealthy food groups, such as fast foods, sweets, and salty snacks, as displayed in Fig. 6.

Respondents in Kenitra and Copenhagen reported the highest intakes of legumes. The most frequent intake of vegetables, nuts and whole-grain bread was reported by respondents in Copenhagen (Figs. 4 and 5). In Kenitra, the most frequent intake of various animal proteins (e.g., fish, milk, dairy products, eggs, white and red meats) (Figs. 4 and 5), sugary drinks, fast foods, and salty snacks (Fig. 6) was observed. Respondents in Kenitra also indicated the highest intake of nuts, whole-grain bread, whole-grain cereal products, and potatoes.

In North Hessia, a pattern similar to the other CSTs was observed concerning whole-grain cereals (Fig. 4), and the highest consumption of cheese (Fig. 5) and sweets (Fig. 6) was assessed. At the same time, this CST exhibited the least frequent intake of meats, including fish, white meats, red meats (Fig. 5), and processed meats (Fig. 6). In contrast, Warsaw and Cilento respondents reported highest intakes of processed meats (Fig. 6).

## Discussion

The United Nations’ Food and Agriculture Organisation (FAO) and the World Health Organisation (WHO) define sustainable healthy diets as those that cover “all dimensions of an individual´s health and wellbeing, have low environmental pressure and impact; are accessible, affordable, safe and equitable; and are culturally acceptable” [27]. The SHDI provides an indication of the dietary impact on both health and the environment, although we cannot claim with certainty that people with a high SHDI score comply with a healthy and sustainable diet, as some sustainability aspects of the social dimensions are missing, such as wellbeing and accessibility, and on the economic domain, affordability of healthy and sustainable food.

### Regional differences of the SHDI

Gender significantly (*p* < 0.001) influenced diet quality in two CSTs. In North Hessia, males had better diets, while in Warsaw, females had healthier and more sustainable diets. Literature [9, 11] shows that females exhibit healthier behaviour most frequently. Previous studies have also shown that higher education and income are predictors of a better diet [11, 12]. Higher income and education (cf. Table 5) were associated with more sustainable and healthier diets in Warsaw (*p* < 0.05) while no other associations were observed in the remaining CSTs. The fact that not all territories had significant results and/or showed contrasting behaviour to previous studies supports Irz and colleagues [10], who found regional differences beyond national results in terms of diet quality. Overall, respondents in Copenhagen and Warsaw seem to have more sustainable and healthier diets than in the remaining CSTs.

### Dietary intake

In Copenhagen and North Hessia, we observed a notably high consumption of legumes,^1^ with average daily consumption of 29 g and 19 g, respectively. Conversely, meat consumption, specifically red meat, was relatively low, averaging 19 g per day in Copenhagen and 23 g per day in North Hessia. These findings indicate a higher legume and lower red meat consumption compared to national averages, aligning more closely with the recommendations of the PHD [13], which suggests a daily intake of 150 g of legumes and a maximum of 14 g of red meat (cf. Table 2). Our data cannot confirm whether the higher intakes of legumes replace meat protein. In Kenitra, where a high proportion of legumes are included in many traditional dishes [24], a slightly higher intake of pulses, averaging more than 24 g daily, was expected. This was not the case, which could be explained by survey participants potentially recognising meat intake as a symbol of status [28–30] or interpreting meat as part of a diversified diet. Furthermore, other food groups, such as cereals, vegetables and fruits, are cheaper. The Moroccan Nutrition Guide recommends an increase in meat consumption [31], a guideline primarily intended for health professionals, with the assumption that it will be further communicated to consumers.

According to national studies, legumes are consumed in very small quantities in Denmark (just 2 g per day) [32]. However, in the SysOrg survey, Copenhagen showed the highest frequency of legume consumption, followed by Cilento and Kenitra, which corresponds to national intake data [33, 34]. The most frequent intake of red meats was observed in Cilento and Kenitra, while respondents from Kenitra also reported the most frequent intake of other meats. These results were unexpected, as national intake data show a higher intake of meats in Denmark [35] and Germany [36]. Cilento exhibited the lowest vegetable intake, with an average of 84 g per day, significantly below the PHD recommendation of 300 g per day. This was unexpected, given the region's alignment with traditional Mediterranean dietary patterns and the likely availability of fresh, local produce [37].

### Strengths and limitations

The investigation has several strengths and limitations. One of the key strengths of this study is its use of the dietary index (SHDI), which allowed for the classification of dietary quality across different populations based on the globally applicable reference diet, PHD [2]. The PHD was chosen as a science-based target for the investigation of diet quality as it takes into considerations both health and environmental impacts. This dual focus is a significant strength in comprehensively assessing dietary patterns. However, the integration of both aspects prevents the possibility of distinguishing between them separately. The survey was adapted to local circumstances; questions were culturally appropriate and understandable by locals. Other dietary indexes, such as SHED, WISH, PHDI [13, 14, 38] also use the PHD as a reference and applied it to extensive dietary intake data. The SHDI uses a simpler food frequency questionnaire of 24 food groups, proposing an assessment that can be easily applied in other thematic surveys and is economically feasible.

Second, the food group level allows for the selection of culture-specific foods, potentially improving respondents’ ability to identify the food groups in their own meals. Moreover, the food group approach facilitates dietary intake assessment as it reduces the number of questions and is relatable to meals and enables the context of cultural circumstances. This methodology could be validated against established dietary intake assessment tools for future research. As with other assessment methods [39], the intake of food groups is based on human estimations and can over- or underestimate the real intake. This also includes the fact that food groups may be perceived and interpreted differently, for instance, what wholegrain foods are. A third aspect that should be discussed is the sampling methods. As the sampling methods differed between the five CSTs, part of the measured differences across CSTs may be due to these sampling differences. The SysOrg study aimed for a unified sampling method across all five CSTs; however, two rural areas demanded additional sampling approaches to obtain a sufficient number of respondents. Other scholars have addressed the difficulty of obtaining identical sampling designs across nations [40]. Participation in the survey was voluntary, and river sampling was used as a non-probabilistic method, which does not give a representative picture of the populations. Online surveys tend to be biased as they may attract primarily people who have an interest in food and exclude people who are less health conscious or less familiar with digital media and tools. Lastly, we acknowledge that the PHD, despite being designed to be universally applicable, might not be appropriate to all populations. Different studies have proposed versions of the PHD adapted to local dietary habits, such as in Chile [41], Denmark [25] and Taiwan [42]. However, for the purpose of a cross-country comparison, the PHD was deemed the most suitable and universally applicable reference diet.

## Conclusions

The SysOrg survey examined the diet quality of 2,210 respondents across five CSTs. This study shows the benefits of using the PHD with common global reference values instead of different national dietary recommendations to evaluate healthy and sustainable diets across diverse regions. The SHDI is a useful tool to score region-specific trends based on a food-group frequency questionnaire. The SHDI covers several domains of sustainable and healthy diets [27] but it cannot stand alone and demands complementary studies on certain aspects of the social and economic domains.

There is an urgent need for current food systems to transition to more sustainable and resilient systems and meet the purposes of the SDGs [5]. Some regions in our study show beneficial eating patterns, for instance, legume intake in Copenhagen, Kenitra and Cilento. Meat continues to be an overconsumed food in most CSTs. The perception of meat consumption as a status symbol of wealth could pose health risks, underscoring the need for targeted interventions. Our study highlights the need for further research on promoting cultural acceptance of sustainable and healthier dietary behaviours, such as increasing vegetable intake and reducing meat consumption.

## Supplementary Information

Below is the link to the electronic supplementary material.

Supplementary file1 (DOCX 349 kb)

## Footnotes

1 In the SysOrg survey, participants were asked about the frequency of consumption of a specific food, assumed to be legumes that were ready to eat (i.e., soaked and cooked). For consistency, we used the cooked weight of the portions when calculating the Sustainable Healthy Diet Index (SHDI).
