# Supplementary material for: Sustainable and healthy diet index (SHDI) unveils regional differences in Europe and Northern Africa: findings from the SysOrg study
Source: Eur J Nutr. 2025 Sep 11;64(6):275. doi: 10.1007/s00394-025-03793-9 (PMC12423132; doi:10.1007/s00394-025-03793-9)

**Supplementary Material (SM)**

**SM 1: SysOrg Household Survey – Selected Questions of Part 1 and 2 Used for this Study**

| **Part 1 - Sociodemographic information** | | | | | | | | | |
| --- | --- | --- | --- | --- | --- | --- | --- | --- | --- |
| **The questionnaire should be filled out by the person primarily responsible for preparing and purchasing food in your household.** | | | | | | | | | |
| 1. **Do you live in this territory [TERRITORY NAME]?** | | | | | | | | | |
| Yes | | | | | No [survey cut-off] | | | | |
| 1. **Which municipality/administrative part of the city/region (district) do you live in?** | | | | | | | | | |
| Name: _____________________________ Zip Code: ________________________________ | | | | | | | | | |
| 1. **Your age (in years)** | | | | | | | | | |
|  | | | | | | | | | |
| 1. **Your gender*** | | | | | | | | | |
| Female | Male | | | No pronoun | | | | I prefer not to answer | |
| 1. **Your level of education**** | | | | | | | | | |
| No formal education | | Primary education (1-4 years) | Lower secondary education (5-10 years) | | | | Upper secondary education (10-13 years) | | |
| Apprenticeship (2-3 years) | | Bachelor’s degree or equivalent level (3 years) | Master’s degree or equivalent level (e.g. Diploma) (3+2 years) | | | | Doctoral studies (PhD) and/or higher | | |
| 1. **Household members** | | | | | | | | | |
| Total number of household members | | | | | | | | | |
| Age in Years | | Female Members | | | | Male Members | | | Other |
| < 1 | |  | | | |  | | |  |
| 1-9 | |  | | | |  | | |  |
| 10-17 | |  | | | |  | | |  |
| Adults (≥18) | |  | | | |  | | |  |
| 1. **Disposable Net Household Income (in Euro) per year**** | | | | | | | | | |
| Up to 18.000 | | 18.001-27.000 | | | | 27.001-36.000 | | | 36.001-46.000 |
| 46.001-57.000 | | 57.001-72.000 | | | | More than 72.000 | | | I prefer not to answer |

| **Part 2 - Diet** | | | | | | | | | | | | | | | | | | | | | | | | | | | | | |
| --- | --- | --- | --- | --- | --- | --- | --- | --- | --- | --- | --- | --- | --- | --- | --- | --- | --- | --- | --- | --- | --- | --- | --- | --- | --- | --- | --- | --- | --- |
| 1. **How often do you eat fruits (excluding dried fruits)?** (*Tick one box*) | | | | | | | | | | | | | | | | | | | | | | | | | | | | | |
| Never | Less than once a month | | | 1-3 times per month | | | Once a week | | | | 2-4 times per week | | | 5-6 times per week | | | Once a day | | | 2-3 times per day | | | | 4-5 times per day | | | | Every time I eat | |
| 1. **How often do you eat dried fruits?** (*Tick one box*) | | | | | | | | | | | | | | | | | | | | | | | | | | | | | |
| Never | Less than once a month | | | 1-3 times per month | | | Once a week | | | | 2-4 times per week | | | 5-6 times per week | | | Once a day | | | 2-3 times per day | | | | 4-5 times per day | | | | Every time I eat | |
| 1. **How often do you eat vegetables (excluding potatoes)?** (*Tick one box*) | | | | | | | | | | | | | | | | | | | | | | | | | | | | | |
| Never | Less than once a month | | | 1-3 times per month | | | Once a week | | | | 2-4 times per week | | | 5-6 times per week | | | Once a day | | | 2-3 times per day | | | | 4-5 times per day | | | | Every time I eat | |
| 1. **How often do you eat legumes (e.g. beans, peas, lentils)?** (*Tick one box*) | | | | | | | | | | | | | | | | | | | | | | | | | | | | | |
| Never | Less than once a month | | | 1-3 times per month | | | Once a week | | | | 2-4 times per week | | | 5-6 times per week | | | Once a day | | | 2-3 times per day | | | | 4-5 times per day | | | | Every time I eat | |
| 1. **How often do you eat non-processed nuts, including peanuts (e.g. unsalted, non-roasted, not sugar-coated)?** *(Tick one box)* | | | | | | | | | | | | | | | | | | | | | | | | | | | | | |
| Never | Less than once a month | | | 1-3 times per month | | | Once a week | | | | 2-4 times per week | | | 5-6 times per week | | | Once a day | | | 2-3 times per day | | | | 4-5 times per day | | | | Every time I eat | |
| 1. **How often do you eat whole-grain bread?** (*Tick one box*) | | | | | | | | | | | | | | | | | | | | | | | | | | | | | |
| Never | Less than once a month | | | 1-3 times per month | | | Once a week | | | | 2-4 times per week | | | 5-6 times per week | | | Once a day | | | 2-3 times per day | | | | 4-5 times per day | | | | Every time I eat | |
| 1. **How often do you eat white bread?** (*Tick one box*) | | | | | | | | | | | | | | | | | | | | | | | | | | | | | |
| Never | Less than once a month | | | 1-3 times per month | | | Once a week | | | | 2-4 times per week | | | 5-6 times per week | | | Once a day | | | 2-3 times per day | | | | 4-5 times per day | | | | Every time I eat | |
| 1. **How often do you eat other non-whole grain cereal products (e.g. pasta, rice)??** (*Tick one box*) | | | | | | | | | | | | | | | | | | | | | | | | | | | | | |
| Never | Less than once a month | | | 1-3 times per month | | | Once a week | | | | 2-4 times per week | | | 5-6 times per week | | | Once a day | | | 2-3 times per day | | | | 4-5 times per day | | | | Every time I eat | |
| 1. **How often do you eat other whole grain cereal products (e.g. pasta, rice)?** (*Tick one box*) | | | | | | | | | | | | | | | | | | | | | | | | | | | | | |
| Never | Less than once a month | | | 1-3 times per month | | | Once a week | | | | 2-4 times per week | | | 5-6 times per week | | | Once a day | | | 2-3 times per day | | | | 4-5 times per day | | | | Every time I eat | |
| 1. **How often do you eat potatoes?** (*Tick one box*) | | | | | | | | | | | | | | | | | | | | | | | | | | | | | |
| Never | | Less than once a month | | | | 1-3 times per month | | | Once a week | | | | 2-4 times per week | | | 5-6 times per week | | | Once a day | | | | 2-3 times per day | | | 4-5 times per day | | | Every time I eat |
| 1. **How often do you eat white meat (e.g. rabbit, chicken, turkey, other poultry)?** (*Tick one box*) | | | | | | | | | | | | | | | | | | | | | | | | | | | | | |
| Never | Less than once a month | | | 1-3 times per month | | | Once a week | | | | 2-4 times per week | | | 5-6 times per week | | | Once a day | | | 2-3 times per day | | | | 4-5 times per day | | | | Every time I eat | |
| 1. **How often do you eat red meat (e.g. beef, pork, veal, lamb)?** (*Tick one box*) | | | | | | | | | | | | | | | | | | | | | | | | | | | | | |
| ☐Never | ☐ Less than once a month | | | ☐1-3 times per month | | | ☐Once a week | | | | ☐2-4 times per week | | | ☐5-6 times per week | | | ☐Once a day | | | ☐2-3 times per day | | | | ☐4-5 times per day | | | | ☐Every time I eat | |
| 1. **How often do you eat processed meat (e.g. cured ham and turkey, salami)? (Tick one box)** | | | | | | | | | | | | | | | | | | | | | | | | | | | | | |
| Never | Less than once a month | | | 1-3 times per month | | | Once a week | | | | 2-4 times per week | | | 5-6 times per week | | | Once a day | | | 2-3 times per day | | | | 4-5 times per day | | | | Every time I eat | |
| 1. **How often do you eat fish or shellfish, including processed fish (e.g. canned tuna, smoked salmon)?** (*Tick one box*) | | | | | | | | | | | | | | | | | | | | | | | | | | | | | |
| Never | Less than once a month | | | 1-3 times per month | | | Once a week | | | | 2-4 times per week | | | 5-6 times per week | | | Once a day | | | 2-3 times per day | | | | 4-5 times per day | | | | Every time I eat | |
| 1. **How often do you eat/drink dairy products (excluding cheese) (e.g. milk, yogurt, buttermilk, kefir , skyr and other fermented dairy products)?** (*Tick one box*) | | | | | | | | | | | | | | | | | | | | | | | | | | | | | |
| Never | Less than once a month | | | 1-3 times per month | | | Once a week | | | | 2-4 times per week | | | 5-6 times per week | | | Once a day | | | 2-3 times per day | | | | 4-5 times per day | | | | Every time I eat | |
| 1. **How often do you eat cheese?** (*Tick one box*) | | | | | | | | | | | | | | | | | | | | | | | | | | | | | |
| Never | Less than once a month | | | 1-3 times per month | | | Once a week | | | | 2-4 times per week | | | 5-6 times per week | | | Once a day | | | 2-3 times per day | | | | 4-5 times per day | | | | Every time I eat | |
| 1. **How often do you eat butter and/or margarine?** (*Tick one box*) | | | | | | | | | | | | | | | | | | | | | | | | | | | | | |
| ☐Never | ☐ Less than once a month | | | ☐1-3 times per month | | | ☐Once a week | | | | ☐2-4 times per week | | | ☐5-6 times per week | | | ☐Once a day | | | ☐2-3 times per day | | | | ☐4-5 times per day | | | | ☐Every time I eat | |
| 1. **How often do you eat eggs?** (*Tick one box*) | | | | | | | | | | | | | | | | | | | | | | | | | | | | | |
| Never | Less than once a month | | | 1-3 times per month | | | Once a week | | | | 2-4 times per week | | | 5-6 times per week | | | Once a day | | | 2-3 times per day | | | | 4-5 times per day | | | | Every time I eat | |
| 1. **How often do you drink alcohol (e.g. wine, beer)?** (*Tick one box*) | | | | | | | | | | | | | | | | | | | | | | | | | | | | | |
| Never | Less than once a month | | | 1-3 times per month | | | Once a week | | | | 2-4 times per week | | | 5-6 times per week | | | Once a day | | | 2-3 times per day | | | | 4-5 times per day | | | | Every time I eat | |
| 1. **How often are the alcoholic beverages you eat certified organic?***** (*Tick one box*) | | | | | | | | | | | | | | | | | | | | | | | | | | | | | |
| Always | | | Often | | | | | | | Sometimes | | | | | | Rarely | | | | | Never | | | | | | I don’t know | | |
| 1. **How often do you drink sugary drinks (e.g. coke, orange soda) including fruit juices and sugar sweetened tea and coffee?** (*Tick one box*) | | | | | | | | | | | | | | | | | | | | | | | | | | | | | |
| Never | Less than once a month | | | 1-3 times per month | | | Once a week | | | | 2-4 times per week | | | 5-6 times per week | | | Once a day | | | 2-3 times per day | | | | 4-5 times per day | | | | Every time I eat | |
| 1. **How often do you eat fast food (e.g. burgers, shawarma, hot dog or similar)?** (*Tick one box*) | | | | | | | | | | | | | | | | | | | | | | | | | | | | | |
| Never | Less than once a month | | | 1-3 times per month | | | Once a week | | | | 2-4 times per week | | | 5-6 times per week | | | Once a day | | | 2-3 times per day | | | | 4-5 times per day | | | | Every time I eat | |
| 1. **How often do you eat desserts/sweets (e.g. ice cream, cake)?** (Tick one box) | | | | | | | | | | | | | | | | | | | | | | | | | | | | | |
| Never | | Less than once a month | | | 1-3 times per month | | | Once a week | | | | 2-4 times per week | | | 5-6 times per week | | | Once a day | | | | 2-3 times per day | | | 4-5 times per day | | | Every time I eat | |
| 1. **How often do you eat sauce** (incl. hot and cold sauces, e.g. béchamel, béarnaise sauce and ketchup, mayonnaise, cocktail sauce) (*Tick one box*) | | | | | | | | | | | | | | | | | | | | | | | | | | | | | |
| Never | | Less than once a month | | | 1-3 times per month | | | Once a week | | | | 2-4 times per week | | | 5-6 times per week | | | Once a day | | | | 2-3 times per day | | | 4-5 times per day | | | Every time I eat | |
| 1. **How often do you eat processed salty snacks (e.g. nuts, crisps, popcorn, crackers, pretzels)?** (*Tick one box*) | | | | | | | | | | | | | | | | | | | | | | | | | | | | | |
| Never | | Less than once a month | | | 1-3 times per month | | | Once a week | | | | 2-4 times per week | | | 5-6 times per week | | | Once a day | | | | 2-3 times per day | | | 4-5 times per day | | | Every time I eat | |

*Gender: “No pronoun” was not asked in the CST Kenitra (Morocco) based on religious beliefs
**Education and Income: Adaptions made based on the local education system. Income: The average income (middle) was determined, three lower and three higher categories placed; income ranges used in the different CST are displayed on SM3.
***Food groups: Example food groups as an explanation for the food frequency questions indicated as “(e.g. food)”, were culturally adapted to local food habits where necessary

SM 2: Literature research on existing dietary indexes

| Study | Food groups/nutrients | Methods |
| --- | --- | --- |
| Malmo Diet and Cancer cohort  Based on Swedish FBDG | SFA, PUFA, fish and shellfish, dietary fibre, fruit and vegetables, and sucrose | 1 assigned for compliance  0 assigned for no compliance  Total score 0 to 6 |
| Danish adults 2003-2008  Based on Danish FBDG 2005 | %E total fat, %E saturated fat, %E added sugar, cereals intake, fruit and vegetables intake, fish intake | Actual intake/recommended  Or  1-[intake/recommended] |
| Dutch Healthy Diet index 2015  Based on the Dutch FBDG 2015 | All  Adequacy components  Moderation components  Optimum components | 0 to 10 for each food group  No extra points; 20g ethanol/d for females and 30g ethanol/d males |
| Finnish University Students  Based on WHO guidelines | Sweets, cake/cookies, snacks, fast food/canned food, lemonade/soft drinks, number of fruit/vegetables servings per day, dairy, cereals, meats, fish & seafood | Adherence or no adherence depending on the food group  E.g.: Consequently, for the number of daily fruit/vegetables servings, ‘3–4 times’ and ‘≥5 times’ were considered as recommended |
| Dutch sub-cohort of the European Prospective Investigation into Cancer and Nutrition | All | WHO’s Healthy Diet Indicator (HDI)  DASH score  Dutch Healthy Diet index 2015 (DHD15-index) |
| \| NutriNet-Santé study cohort  Org-FFQ (264 items) for intake \| \| --- \| | \| All  Dietary energy balance  Dietary energy density  Dietary diversity index \| \| --- \| | \| Sustainable Diet Index (SDI)  Diet + environmental + economic + sociocultural aspects \| \| --- \| |
| \| NND adherence Norway \| \| --- \| | \| Nordic foods  Meal pattern, Nordic fruits, root vegetables, cabbages, potatoes vs rice/pasta, whole-grain vs white bread, oatmeal, foods from the wild countryside, milk vs juice, water vs sweetened beverages \| \| --- \| | \| NND score \| \| --- \| |
| \| The SHED Index \| \| --- \| | \| All  + eating behaviours + purchase behaviours \| \| --- \| | \| The EAT-Lancet reference diet and the Mediterranean Diet score \| \| --- \| |
| \| WISH  Globally applicable \| \| --- \| | All | \| The EAT-Lancet reference diet \| \| --- \| |
| \| EPIC-Postdamstudy \| \| --- \| | \| NND: whole-grain and rye bread, berries, apple & pear, fish, cabbages, root vegetables, dairy, potatoes, fats  MedDiet: cereals, fruits & nuts, vegetables, fish, legumes, meat, dairy, alcohol, olive oil \| \| --- \| | \| NND score  MedDiet score \| \| --- \| |

SM 3: Household income groups of the five case study territories: Range converted to Euro and calculated mean values

|  | **Cilento (Italy)** | | **Copenhagen (Denmark)** | | **North Hesse (Germany)** | | **Kenitra (Morocco)** | | **Warsaw (Poland)** | |
| --- | --- | --- | --- | --- | --- | --- | --- | --- | --- | --- |
|  | Range given in the survey | Mean calculated for analysis | Range given in the survey | Mean calculated for analysis | Range given in the survey | Mean calculated for analysis | Range given in the survey | Mean calculated for analysis | Range given in the survey | Mean calculated for analysis |
| Level 1 | Up to 18000 | 18000 | Up to 18000 | 18000 | Up to 18000 | 18000 | Up to 3400 | 3400 | Up to 6450 | 6450 |
| Level 2 | 18001-27000 | 22500.5 | 18001-30000 | 24000.5 | 18001-27000 | 22500.5 | 3401-5600 | 4500.5 | 6451-9765 | 8108 |
| Level 3 | 27001-36000 | 31500.5 | 30001-47000 | 38500.5 | 27001-36000 | 31500.5 | 5601-8400 | 7000.5 | 9766-12900 | 11333 |
| Level 4 | 36001-46000 | 41000.5 | 47001-57000 | 52000.5 | 36001-46000 | 41000.5 | 8401-11200 | 9800.5 | 12901-16555 | 14728 |
| Level 5 | 46001-57000 | 51500.5 | 57001-67000 | 62000.5 | 46001-57000 | 51500.5 | 11201-14000 | 12600.5 | 16556-20640 | 18598 |
| Level 6 | 57001-72000 | 64500.5 | 67001-77000 | 72000.5 | 57001-72000 | 64500.5 | 14000-16900 | 15450 | 20641-25800 | 23220.5 |
| Level 7 | More than 72000 | 108000 | More than 77000 | 115500 | More than 72000 | 108000 | More than 16900 | 25350 | More than 25800 | 38700 |

Danish krone= 0.133 EU. Moroccan dirham= 0.095 EU. Polish zloty = 0.215 EU.

Calculations of the mean:

Level 1 was based on upper value.
Level 2 to 6 was based on the mean of the range (Eq(1)).
Level 7 was calculated based on the mean of the value and an assumed double value (Eq (2)).

Eq(1): $\frac{(\text{Lower Value }+ \text{Upper Value})}{2}$

Example Eq(1)_level2_CI: $\frac{(\text{18001}+\text{27000})}{2}$ = 22500.5

Eq(2): $\frac{(\text{Upper Value }+ (\text{Upper Value*2)})}{2}$

Example Eq(2)_level7_CI: $\frac{(\text{72000}+(\text{72000*2)})}{2}$ = 108000


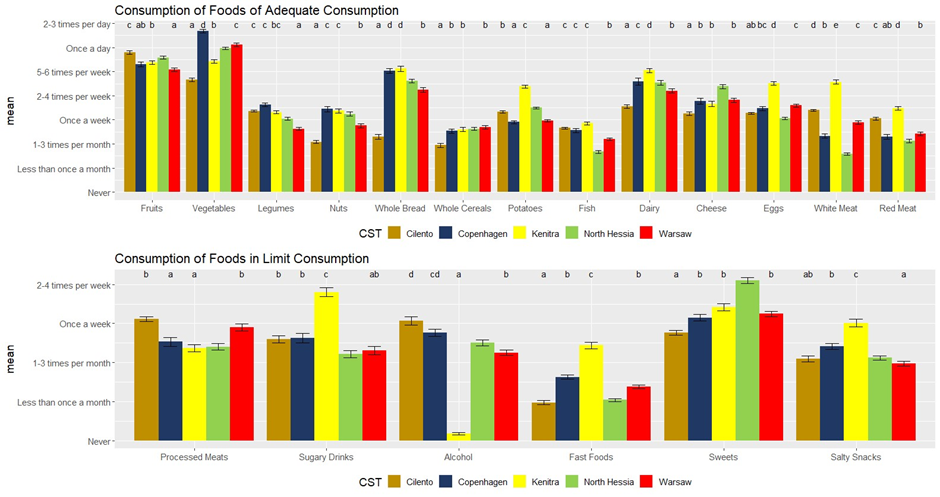

Supplement: Supplementary file 1 — Supplementary file1 (DOCX 349 kb) [file 394_2025_3793_MOESM1_ESM.docx]
